# Long-Term Outcome of Bypass Surgery versus Endovascular Revascularization in Long Femoropopliteal Lesions

**DOI:** 10.3390/jcm12103507

**Published:** 2023-05-17

**Authors:** Michaela Kluckner, Leonhard Gruber, David Wippel, Daniela Lobenwein, Werner Westreicher, Manuela Pilz, Florian K. Enzmann

**Affiliations:** 1 Department of Vascular Surgery, Medical University Innsbruck, 6020 Innsbruck, Austria; michaela.kluckner@i-med.ac.at (M.K.); david.wippel@i-med.ac.at (D.W.); werner.westreicher@i-med.ac.at (W.W.); 2 Department of Radiology, Medical University Innsbruck, 6020 Innsbruck, Austria; leonhard.gruber@i-med.ac.at; 3 Department of Cardiac, Vascular and Endovascular Surgery, Paracelsus Medical University Salzburg, 5020 Salzburg, Austria; m.aspalter@salk.at

**Keywords:** prosthetic graft, vein graft, nitinol stent, bypass, peripheral arterial disease, patency rates, chronic limb threatening ischemia, long-term follow-up

## Abstract

Long-term follow-up data comparing surgical and endovascular revascularization of femoropopliteal lesions is rarely reported. This study presents 4-year results of revascularization for long femoropopliteal lesions (Trans-Atlantic Inter-Society Consensus Types C and D) with vein bypass (VBP), polytetrafluorethylene bypass (PTFE), and endovascular intervention with a nitinol stent (NS). Data from a randomized-controlled trial on VBP and NS was compared with a retrospective patient cohort using PTFE with the same inclusion and exclusion criteria. Primary, primary assisted, and secondary patency, as well as changes in Rutherford categories and limb salvage rates, are reported. Between 2016 and 2020, 332 femoropopliteal lesions underwent revascularization. The lesion lengths and basic patient characteristics were similar between the groups. 49% of the patients presented with chronic limb threatening ischemia at the time of revascularization. During the four-year follow-up, primary patency was comparable for all three groups. Primary assisted and secondary patency were significantly higher after VBP, while PTFE and NS had similar results. Clinical improvement was also significantly superior after VBP. After four years of follow-up, patency rates as well as the clinical outcome clearly favor VBP. If no vein is available, NS is as effective as PTFE bypass with regard to patency and clinical outcome.

## 1. Introduction

Guidelines on the treatment of femoropopliteal lesions in patients with peripheral arterial disease have changed over the years. Early recommendations of an endovascular-first approach only in short lesions have been altered to a broader recommendation of endovascular treatment (EVT) for femoropopliteal lesions up to 25 cm in the current European Society of Vascular Medicine (EVSM) guidelines [1]. This trend towards EVT is further emphasized by a recent meta-analysis of five randomized controlled trials (RCT) comparing bypass surgery with EVT of femoropopliteal lesions. At two years, similar major adverse limb events (MALE) and amputation-free survival were reported, with significantly lower rates of complications and shorter hospital stays after EVT [2].

In contrast, the BEST-CLI trial reported a clear advantage of vein bypass surgery (VBP) compared to EVT in patients with chronic limb-threatening ischemia (CLTI) [3]. Regarding the choice of graft material for bypass surgery, only moderate- to low-quality data is available [4]. However, most guidelines recommend the greater saphenous vein (GSV) as the conduit of choice for femoropopliteal bypass surgery. The use of prosthetic graft material such as polytetrafluorethylene (PTFE) should be limited to above-knee revascularizations and only used in cases of absent autologous vein grafts [5]. In a recent Vascular Quality Initiative study analyzing the impact of graft material on occlusion- and amputation-free survival in above- and below-knee femoropopliteal bypass surgery, single-segment vein bypass showed superior outcome compared to other graft materials after one year of follow-up [6]. However, Dalmia et al. showed only a marginally superior outcome of single-segment vein bypass regarding amputation-free survival and primary patency [7].

Our group previously reported similar 2-year patency rates from pooled data from a RCT and a retrospective cohort of polytetrafluorethylene bypass (PTFE), vein bypass, and angioplasty with nitinol stents (NS) for the treatment of femoropopliteal Trans-Atlantic Inter-Society Consensus (TASC II) type C and D lesions [8,9]. The aim of this study was to compare the outcomes of the PTFE, VBP, and NS groups at four years. 

## 2. Materials and Methods

### 2.1. Study Design

Retrospective data of patients undergoing primary prosthetic bypass surgery for femoropopliteal TASC II C and D lesions at the University Hospital Innsbruck were combined with data from the largest RCT comparing autologous VBP with angioplasty and NS. This was conducted at the University Hospital Salzburg (registered at ISRCTN18315574) [10]. The patients in the retrospective analysis were screened for the same inclusion and exclusion criteria valid for the RCT except for the use of prosthetic graft material, which was an exclusion criterion in the RCT. All patients underwent surgery between March 2016 and July 2020. The follow-up examinations were performed at 3, 6, and 12 months, with the following annual examinations including ankle-brachial index (ABI) and duplex sonography.

Adult patients (≥30 years) with femoropopliteal TASC II C or D lesions with patent ipsilateral iliac arteries and at least one patent tibial artery were included in the study. Severe intermittent claudication (<200 m of walking distance, Rutherford category 3) or CLTI, including rest pain and ischemic lesions (Rutherford categories 4–6), with onset of symptoms at least two months prior, was mandatory for study inclusion.

Exclusion criteria involved embolic or traumatic femoropopliteal occlusions, acute ischemia, any form of vasculitis, or previous ipsilateral bypass surgery. Patients that were considered too frail for surgery (American Society of Anaesthesiologists classification—ASA > 3) as well as pregnant patients were not included in the study.

Primary endpoints were primary and secondary patency. Primary patency was defined as the absence of flow-limiting stenosis (peak systolic velocity ratio > 2.5) or occlusion of the treated vessel. A secondary procedure (e.g., bypass thrombectomy with correction of inflow and outflow or anastomoses, interventional re-canalization, and re-stenting) necessary for graft or stent occlusion in an afterwards patent vessel was classified as secondary patency.

Secondary endpoints included primary assisted patency, target lesion revascularization (TLR), limb salvage, and survival. Primary assisted patency was defined as procedures performed on patent vessels or bypasses in order to prevent occlusions. TLR included procedures due to flow-limiting stenosis or occlusion in the treated segment of the artery. Furthermore, systemic complications (myocardial infarction, postoperative anemia requiring blood transfusion, renal function impairment, sepsis, and stroke), local complications (distal embolization, unplanned minor amputation, pseudo aneurysm, superficial or deep surgical site infection, vessel perforation), survival, and clinical improvement were assessed by the change in Rutherford category [11,12].

### 2.2. Revascularization Protocol

In the preoperative course, antiplatelet therapy with 100 mg acetylsalicylic acid or 75 mg clopidogrel once daily was prescribed. If oral anticoagulation was indicated for other comorbidities, no antiplatelet therapy was given.

In the bypass groups, standard femoropopliteal bypass techniques with proximal and distal end-to-side anastomosis during systemic heparinization (5000 IU) were performed. Antibiotic prophylaxis with a second-generation cephalosporin was administered prior to the skin incision. In cases of stenotic or occluded common femoral arteries (CFA), endarterectomy was carried out prior to the bypass anastomosis. Standard PTFE for supragenicular bypasses and ring-enforced PTFE for infragenicular bypasses (Gore, Flagstaff, AZ, USA) served as graft material in the prosthetic group. If available, the non-reversed GSV was the preferred conduit in the VBP group. Lesser saphenous or arm veins with a diameter of at least 3 mm were used if GSV could not be used. In the postoperative course, 100 mg of acetylsalicylic acid once daily was prescribed to patients without oral anticoagulation for other indications. After infragenicular ePTFE bypass surgery, a lifelong dual antiplatelet therapy with 100 mg acetylsalicylic acid combined with 75 mg clopidogrel was prescribed, except for patients with oral anticoagulation. In these cases, oral anticoagulation in combination with a platelet inhibitor was prescribed. For the endovascular treatment, standard techniques were used with subintimal recanalization, plain balloon angioplasty, and NS (Facile, AMG International, Winsen, Germany, or Pulsar-18, Biotronik, Berlin, Germany) as previously described [10]. In addition to the preoperative medication, clopidogrel 75 mg was prescribed for 6 weeks in the postinterventional course. 

### 2.3. Statistics

All data were stored in Microsoft Excel 16.65 (Microsoft; Redmond, WA, USA). For the statistical analyses, SPSS 27.0 for Windows (SPSS, Chicago, IL, USA), GraphPad Prism 9.4.1 (GraphPad Software LLC; La Jolla, CA, USA), and WEKA 3.8.4 (University of Waikato; Waikato, New Zealand) were used [13].

Continuous data from the groups was compared via an ordinary one-way ANOVA with Tukey multiple-comparison correction, and categorical variables via a χ^2^ test. Invalid or missing data were discarded from further analysis, and no imputation techniques for missing data were applied. Statistical significance was considered for *p*-values < 0.05.

Kaplan-Meier analyses were carried out for primary, primary assisted, and secondary patency rates using a log-rank test to compare curves.

To generate a predictor ranking, the gain ratio feature evaluation with a ranker algorithm was used within WEKA. Results include rank, gain ratio merit, and odds ratios (OR), including 95% confidence intervals (CI), calculated from a binary regression analysis.

## 3. Results

### 3.1. Peri- and Postoperative Results

Between March 2016 and July 2020, 332 extremities in 316 patients (217 males, 69%) underwent revascularization for femoropopliteal TASC II type C and D lesions. Of those cases, 109 extremities (33%, 106 patients) received VBP, 109 lesions were treated with NS (33%, 103 patients), and 114 extremities (34%, 107 patients) underwent PTFE bypass surgery. The clinical characteristics of the patients are given in Table 1. Significantly more patients in the NS group presented with coronary artery disease and subsequent coronary artery bypass compared to the bypass group. Furthermore, significantly higher rates of hypertension and dyslipidemia were detected in this group. All other characteristics were equally distributed between the three groups.

Regarding the ASA classification, an indicator for the patients’ operative risk, the majority of all three treatment groups were classified as ASA 3. The mean lesion lengths were also similar, with no significant differences between the groups (*p* = 0.427); however, significantly more VBP were infragenicular bypasses (VBP 55% vs. PTFE 11%, *p* < 0.001). In the VBP group, significantly more patients underwent previous interventions of the ipsilateral extremity (*p* = 0.004). More than half of the lesions in all three groups were classified as TASC II type D. VBP surgery had significantly longer mean procedure times compared to the NS and PTFE groups (*p* < 0.001) (Table 2). In the postoperative course, significantly higher numbers of superficial and deep surgical site infections (23 (21.1%) vs. 11 (9.6%) cases, *p* < 0.001) as well as blood transfusions (20 (18.3%) vs. 4 (3.5%) cases, *p* < 0.001) were detected in the VBP group compared to the PTFE group. This had an impact on the hospitalization with a significantly longer duration in the VBP group (9 ± 4.3 days vs. NS 3 ± 3.5 days vs. PTFE 6 ± 3.9 days, *p* < 0.001). An overview of all postoperative complications is given in Table 3. 

### 3.2. Patency Rates

**Figure 1:**
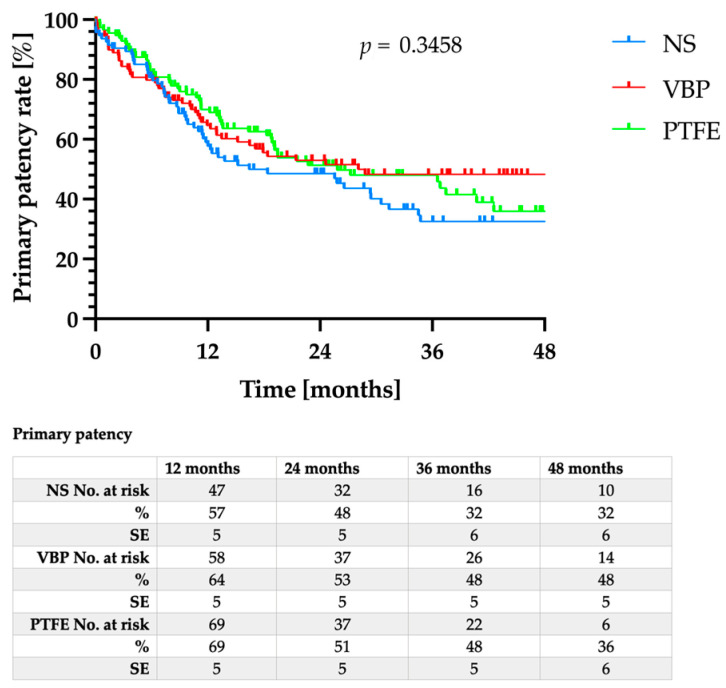
Primary patency rates after nitinol stent (NS) implantation (blue line), vein bypass (VBP, red line), or PTFE bypass (green line).

**Figure 2:**
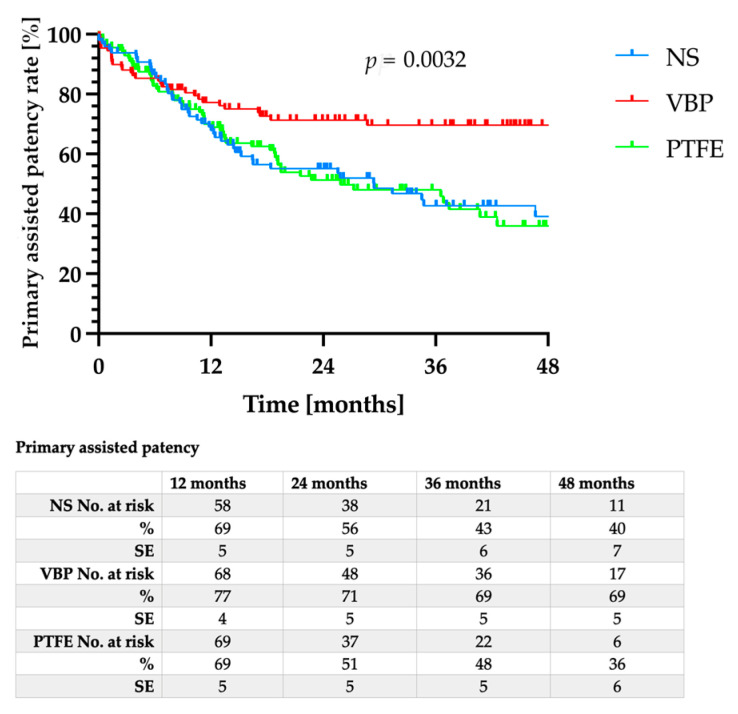
Primary assisted patency after nitinol stent (NS) implantation (blue line), vein bypass (VBP, red line), or PTFE bypass (green line).

**Figure 3:**
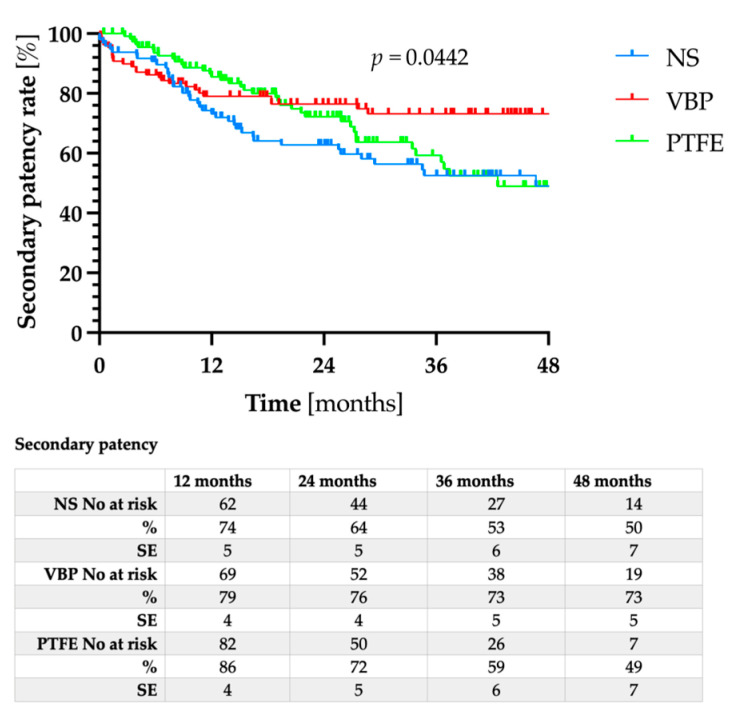
Secondary patency rates after nitinol stent (NS) implantation (blue line), vein bypass (VBP, red line), or PTFE bypass (green line).

The mean follow-up was 32 ± 16.5 months, with no significant differences between the three groups (*p* = 0.137). While the primary patency for VBP did not differ significantly from either NS or PTFE (Figure 1, *p* = 0.346), the primary assisted patency (Figure 2, *p* = 0.003) as well as the secondary patency (Figure 3, *p* = 0.044) for VBP were significantly superior compared to NS and PTFE.

There were no significant differences between the three groups regarding the freedom from TLR (NS 39%, PTFE 35%, VBP 36%; *p* = 0.844) as well as the number of revascularizations (NS *n* = 84, PTFE *n* = 73, VBP *n* = 69, *p* = 0.603) at four years. However, significantly more angioplasties of bypasses were performed in the VBP group (*n* = 31, 28%) compared to the PTFE group (*n* = 8; 7%).

During follow-up, 20 (18%) patients of the NS required bypass surgery, whereas 13 (11%) patients of the PTFE groups and 8 (7%) patients after VBP received a redo-bypass. 

### 3.3. Ranking

A gain ratio merit-based ranking of all included predictors for secondary patency revealed the type of surgery to be the strongest predictor for secondary patency, as confirmed by binary regression analysis (Table 4). Even though other predictors such as high preoperative Rutherford classification, CLTI, or superficial wound infection also demonstrated predictive value, none reached statistical significance.

### 3.4. Clinical Outcome

**Figure 4:**
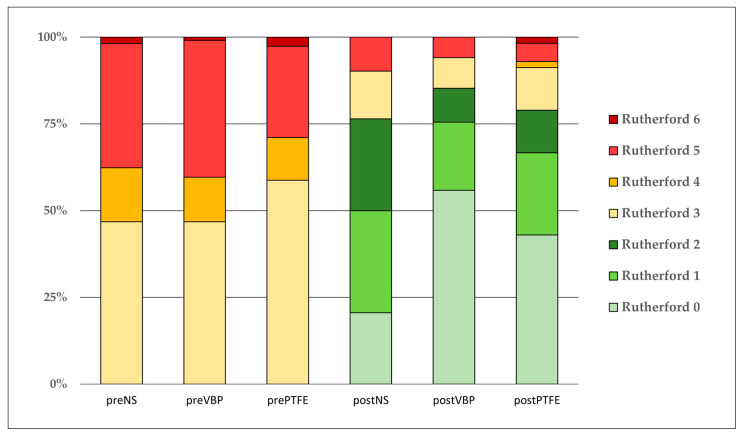
Change of Rutherford categories after revascularization. Distribution of Rutherford categories before the procedures (left) and at the end of follow-up (right), excluding all major amputations.

In total, 49% of the study population presented with CLTI prior to revascularization. In the PFTE group, the majority (59%) of patients suffered from severe lifestyle-limiting claudication, whereas in the VBP and NS groups, CLTI was more frequent without statistical significance (Figure 4). The comparison of the baseline values of the Rutherford categories pre revascularization with the values at the latest follow-up showed improvements in all groups after excluding patients with major amputations. After revascularization, clinical improvement was highest in the VBP group compared to the other groups. The mean change in the Rutherford category was significantly better in the VBP group (3 ± 1.6) compared to the PTFE (2 ± 1.8) and NS group (2 ± 1.5) (*p* = 0.006). In the VBP group, 83% improved at least one Rutherford category after revascularization versus 80% in the PTFE group and 76% in the NS group (*p* = 0.384). Significantly more patients in the bypass groups (VBP as well as PTFE) presented with Rutherford category 0 or 1 compared to the NS group at the end of follow-up (VBP 71%, PTFE 67% vs. NS 47%; *p* < 0.001). No change in the Rutherford category was seen in 16% of the NS group vs. 11% in the VBP and PTFE groups, respectively (*p* = 0.253). A deterioration of the Rutherford category at last follow-up compared to before revascularization was more frequent in the PTFE group (5%) versus the VBP (1%) and NS group (1%) (*p* = 0.054). All patients presenting with Rutherford category 6 prior to revascularization achieved wound healing after the revascularization. Both patients in the PTFE group with Rutherford category 6 at the time of last follow-up deteriorated from categories 5 and 4 pre-revascularization. CLTI patients treated with VBP surgery improved significantly better compared to the PTFE and NS groups (VBP: 4 ± 1.5, PTFE: 3 ± 2.2, NS: 3 ± 1.6 Rutherford categories; *p* = 0.004). The limb salvage rates after 4 years were almost identical (VBP: 92%, PTFE: 92%, NS: 90%; *p* = 0.934). In total, major amputation was necessary in 18 (5%) of the patients, with no significant difference between the three groups (NS 6% vs. VBP 6% vs. PTFE 4%, *p* = 0.797). There was no perioperative mortality, and survival after 4 years was comparable (VBP: 89%, PTFE: 82%, NS: 81%; *p* = 0.634).

## 4. Discussion

Recent publications comparing surgical and endovascular revascularization techniques rarely report follow-up data beyond one or two years [6,14,15,16]. In the current study, retrospectively analyzed 4-year results of patients treated for femoropopliteal TASC II Type C and D lesions with either NS, VBP, or PTFE are presented. Despite the promising results after two years, with similar patency rates between the three groups, the 4-year results showed significantly better primary assisted and secondary patency after VBP compared to PTFE and NS, as well as a significantly better clinical outcome after VBP compared to PTFE and NS.

In the majority of guidelines, the GSV is recommended as the conduit of choice for femoropopliteal bypass surgery; however, prosthetic grafts are a viable alternative for above-knee bypass surgery [1,17]. Criteria in favor of prosthetic grafts are inadequate autologous veins, shorter procedure times, less tissue trauma, and shorter hospitalization [6]. In our cohort, reinterventions for superficial or deep surgical site infections were twice as high in the VBP group compared to the PTFE group, resulting in a significantly longer hospital stay. Postoperative blood transfusions were five times higher in the VBP group compared to the PTFE group. Sparing the GSV for coronary bypass procedures or subsequent distal bypass reconstructions is regularly mentioned in the literature. However, in up to 33% of patients with initially preserved GSV, there is no adequate vein graft available at the time of a secondary bypass [8]. Furthermore, if vein grafts are used primarily, the superior patency rates may render further distal bypass reconstructions unnecessary.

Specific anatomical and patient characteristics are known to influence the patency of vascular reconstructions. The length and complexity of the bypass/endovascular procedure, as well as the distal outflow, are well documented factors. In our patient cohort, the lesion length did not differ between the three groups; however, in the VBP group, significantly more below-knee procedures were performed. In the PTFE group, significantly more patients presented with a stenosis-free three-vessel outflow at the time of revascularization. Despite these optimal conditions for the PTFE group, the primary assisted and secondary patency rates were significantly inferior. In our risk factor evaluation for loss of secondary patency, the type of revascularization procedure was the only significant predictor. 

Endovascular treatment of long and complex femoropopliteal lesions has gained importance in everyday clinical practice due to its less invasive character and shorter hospital stay, especially when considering the multimorbid patient population. Farhan et al. recently presented a meta-analysis on open versus endovascular treatment of symptomatic femoropopliteal lesions with a variety of open and interventional techniques. In this study, EVT and bypass surgery showed no differences in amputation-free survival or major adverse limb events after two years, but primary patency was significantly worse after EVT [2]. The previously published 2-year data of the current patient cohort showed no significant differences in patency rates between the NS, PTFE, and VBP groups [9]. However, after four years, the VBP group had significantly better primary assisted and secondary patency rates compared to the results after PTFE and NS, which were almost identical. 

Despite the important role of patency rates as objective parameters after revascularization, the clinical results are more relevant to the patients. In general, significantly more patients reached an asymptomatic clinical stage after bypass surgery compared to EVT; nevertheless, the best clinical improvement was achieved with VBP. A clinically asymptomatic status after four years was two times more likely in the PFTE group and nearly three times more likely in the VBP group compared to the NS group.

About half of the patients in this study presented with CLTI at the time of the revascularization, and their clinical improvement was superior after VBP compared to PTFE and NS. This is in accordance with the results of the recently published BEST-CLI trial. In BEST-CLI, the composite outcome of MALE and death was significantly better in patients with single-segment GSV compared to EVT. Similarly, the rate of above-ankle amputations was significantly lower in the GSV VBP group. Non-GSV grafts and the endovascular group showed similar results [3]. Patients with CLTI belong to the most vulnerable group of vascular patients. Ninety percent of them suffer from significant coronary artery disease, and the risk of cardiovascular complications in the perioperative course is high [17]. In our cohort, 1.2% of the patients suffered from a myocardial infarction (MI) perioperatively, with no differences between the groups. However, due to the exclusion of patients ranked higher than ASA 3, the MI rate might be distorted. The advantage of shorter procedures and fewer wound healing disorders after PTFE bypass has to be balanced against the need for reinterventions due to graft occlusions. 

Our results of a mixed cohort with claudicants and CLTI patients as well as the BEST-CLI trial indicate that patients with longer life expectancy clearly benefit from VBP irrespective of their clinical status prior to the revascularization, despite more postoperative complications and longer hospitalization. If no GSV is available, patency rates as well as the clinical outcome are similar after NS and PTFE, with a tendency towards more clinically asymptomatic patients in the PTFE group. In these cases, the choice of the revascularization method must be an individual one with consideration of the comorbidities of the patients as well as their clinical stage and expectation of clinical improvement. 

### Limitations

In this study, the data from an RCT is compared with retrospectively collected data. The inclusion is limited by the criteria of the RCT, resulting in a highly selective patient cohort. Furthermore, the collection of the included patients was based on anatomical criteria, resulting in a mixed cohort of claudicants and CLTI patients. The relatively small number of patients in each group prohibits further subgroup analysis and might lower the power of the risk factor prediction analysis. The number of patients presenting in Rutherford category 6 is also very low, so the results of this subgroup might be biased with limited generalizability. Despite no significant differences in lesion length between the groups, significantly more below-knee bypasses were performed in the VBP group, limiting the comparability of the results.

## 5. Conclusions

In patients with long femoropopliteal lesions and a life expectancy beyond 2 years, patency rates as well as the clinical outcome favor VBP as the revascularization method of choice, despite more complications in the perioperative period. The results of the NS and PTFE groups were very similar, suggesting an endovascular-first strategy for all long femoropopliteal lesions if no vein graft is available.

## Figures and Tables

**Table 1:** Clinical characteristics.

	Stent (*n* = 103, 109 Lesions)	VBP (*n* = 106, 109 Lesions)	PTFE (*n* = 107, 114 Lesions)	*p* Value ^$^
Age (years)	69.3 ± 7	68.5 ± 7.8	68.7 ± 9.4	0.990 ^◊^
Male	69 (67)	78 (74)	70 (65)	0.269 ^§^
Cardiovascular risk factors				
Atrial fibrillation	15 (15)	15 (14)	11 (10)	0.527 ^§^
Coronary artery bypass	15 (14)	3 (3)	11 (10)	0.014 ^§^
Coronary artery disease	48 (47)	31 (29)	36 (34)	0.033 ^§^
Diabetes mellitus	36 (35)	40 (38)	34 (32)	0.691 ^§^
Dyslipidemia	63 (61)	69 (65)	38 (36)	<0.001 ^§^
Hemodialysis	3 (3)	3 (3)	-	0.138 ^§^
Hypertension	89 (86)	88 (83)	77 (72)	0.033 ^§^
Prior stroke	12 (12)	10 (9)	9 (8)	0.667 ^§^
Rutherford category				0.361 ^§^
3	51 (47)	51 (47)	60 (56)	
4	17 (16)	14 (13)	14 (13)	
5	39 (36)	43 (40)	29 (27)	
6	2 (2)	1 (1)	3 (3)	
CLTI	58 (53)	58 (53)	46 (43)	0.116 ^§^
ASA classification				0.787 ^§^
I	1 (1)	1 (1)	1 (1)	
II	25 (24)	29 (27)	31 (29)	
III	77 (75)	76 (72)	74 (69)	

Values are *n* (%) or mean ± standard error (SE). ABI—ankle-brachial index; ASA—American Society of Anesthesiology; CLTI—chronic limb-threatening ischemia. ^$^ group comparisons: NS vs. VBP/NS vs. PTFE/VBP vs. PTFE. ^§^ χ^2^ test, ^◊^ ordinary one-way ANOVA with Tukey multiple-comparisons correction.

**Table 2:** Procedural findings and lesion characteristics.

	Stent (*n* = 109)	VBP (*n* = 109)	PTFE (*n* = 114)	*p* Value
Lesion length (mm)	264 ± 59	272 ± 70	274 ± 58	0.427 ^◊^
TASC II type				
C	48 (44)	48 (44)	51 (45)	0.993 ^§^
D	61 (56)	61 (56)	63 (55)	0.993 ^§^
Previous intervention ipsilateral	31 (28)	49 (45)	29 (25)	0.004 ^§^
Chronic total occlusion	87 (80)	96 (88)	107 (94)	0.007 ^§^
Reference vessel diameter (mm)	5 ± 0.7	5 ± 1	5.1 ± 1.1	0.783 ^◊^
Number of stenosis free outflow vessels				
1	26 (24)	21 (19)	20 (18)	0.482
2	53 (49)	54 (50)	44 (39)	0.188
3	30 (30)	34 (31)	50 (44)	0.026
CFA endarterectomy	-	39 (36)	33 (29)	<0.001 ^§^
Infragenicular bypass	-	60 (55)	12 (11)	<0.001 ^§^
ePTFE graft	-	-	12 (11)	
Ipsilateral GSV	-	100 (92)	-	
Alternative vein graft	-	9 (8)	-	
Reversed vein graft	-	15 (14)	-	
Stent diameter (mm) *	6 ± 0.5	-	-	
Stented lesion length (mm) *	248 ± 98	-	-	
Number of stents *	4 ± 2	-	-	
CFA angioplasty	4 (4)	-	-	
Popliteal stenting *	30 (28)	-	-	
Procedural length	72 ± 31	181 ± 63	142 ± 54	*p* < 0.001

Values are *n* (%) or mean ± SE. CFA = common femoral artery. ePTFE—ring enforced PTFE; GSV—greater saphenous vein; TASC—TransAtlantic Inter-Society Consensus; * Nitinol stents (Facile, AMG International, Winsen, Germany, or Pulsar-18, Biotronik, Berlin, Germany) with up to 10 cm in length. ^§^ χ^2^ test, ^◊^ ordinary one-way ANOVA with Tukey multiple-comparisons correction.

**Table 3:** Postoperative complications.

	Stent (*n* = 103; 109 Lesions)	VBP (*n* = 106; 109 Lesions)	PTFE (*n* = 107; 114 Lesions)	*p* Value
Patients with ≥ 1 complication	7 (6)	12 (11)	1 (1)	0.007 ^§^
Local complications				
Superficial SSI	-	14 (13)	8 (7)	0.001 ^§^
Deep SSI	-	9 (8)	3 (3)	0.004 ^§^
Bleeding/pseudoaneurysm	6 (6)	6 (6)	3 (3)	0.543 ^§^
Distal embolization	7 (7)	-	-	
Vessel perforation	10 (9)	-	-	
Unplanned minor amputation	4 (4)	1 (1)	1 (1)	0.080 ^§^
Systemic complications				
Anemia/blood transfusion	5 (5)	20 (18)	4 (4)	<0.001 ^§^
Myocardial Infarction	1 (1)	1 (1)	2 (2)	0.797 ^§^
Renal function deterioration	3 (3)	4 (4)	-	0.140 ^§^

Values are *n* (%); SSI—surgical site infection. ^§^ χ^2^ test.

**Table 4:** Gain ratio merit-based predictor ranking for secondary patency.

Predictor	Rank	Gain Ratio Merit	OR [95% CI]	*p*-Value
Surgery type Stent (vs. VBP) PFTE (vs. VBP)	1.7 ± 0.6	0.02 ± 0.003	0.532 [0.302 to 0.938] 0.324 [0.176 to 0.595]	0.001 0.029 0.000
Rutherford classification 5/6	2.8 ± 0.92	0.016 ± 0.002	0.742 [0.344 to 1.604]	0.448
CLTI	3.9 ± 0.94	0.014 ± 0.002	0.726 [0.356 to 1.480]	0.378
Superficial surgical site infection	5.4 ± 1.64	0.011 ± 0.003	0.739 [0.252 to 2.170]	0.582
Atrial fibrillation	5.8 ± 1.83	0.01 ± 0.003	0.620 [0.289 to 1.331]	0.220
Diabetes mellitus	6.9 ± 1.44	0.009 ± 0.002	0.719 [0.425 to 1.216]	0.219
Hypertension	7.5 ± 1.87	0.008 ± 0.002	1.559 [0.824 to 2.950]	0.172

CLTI—chronic limb threatening ischemia.
